# Successful treatment of a rare giant right atrium aneurysm in an 8-year-old child: a case report

**DOI:** 10.1186/s43044-025-00643-1

**Published:** 2025-05-27

**Authors:** Ekaterina Amansakhatova, Timur Khapaev, Ivan Murashov, Serezha Manukian, Dariya Pogrebnitckaya, Yuriy Kulyabin, Bahram Kamangari, Ilya Soynov

**Affiliations:** 1 Meshalkin National Medical Research Center, Novosibirsk, Russian Federation; 2 Novosibirsk State Medical University, Novosibirsk, Russian Federation

**Keywords:** CHD, Right atrial aneurysm, Aneurysm

## Abstract

**Background:**

Among the various paediatric congenital heart disease pathologies, aneurysm of the right atrium represents a relatively uncommon condition and is characterised by a variety of progressive rhythm irregularities. While a number of cardiac surgical institutions advocate for medication in such cases, others persist in supporting radical surgical correction.

**Case presentation:**

This paper presents a clinical case in which radical surgical correction was successfully performed on a rare large aneurysm of the right atrium, associated with supraventricular tachycardia, in an 8-year-old patient.

**Conclusions:**

An aneurysm of the RA is an extremely rare cardiac anomaly with a variable clinical presentation. Despite the availability of a conservative treatment approach, surgical correction remains the preferred option at the majority of cardiac surgery centres. This is not only to prevent the development of complications but also to achieve successful correction of RA aneurysms.

## Background

Among the various paediatric CHD pathologies, aneurysm of the right atrium (RA) represents a relatively uncommon condition. In the majority of cases, the precise aetiology of this anomaly remains undetermined. However, it can be postulated that this defect is the consequence of dysplasia of the muscular wall of the RA. Despite the fact that individuals with an RA aneurysm have a long-term asymptomatic history, it is discovered unexpectedly during preventative screenings. Despite the elevated risk of developing cardiac arrhythmias, which can lead to complications such as pulmonary embolism (PE) and aneurysm rupture, as well as sudden cardiac death, there is a lack of consensus among cardiac surgery centres regarding the optimal treatment for this pathology. This paper presents a clinical case study of the treatment of an 8-year-old child with a giant aneurysm.

## Case Report

**Fig. 1:**
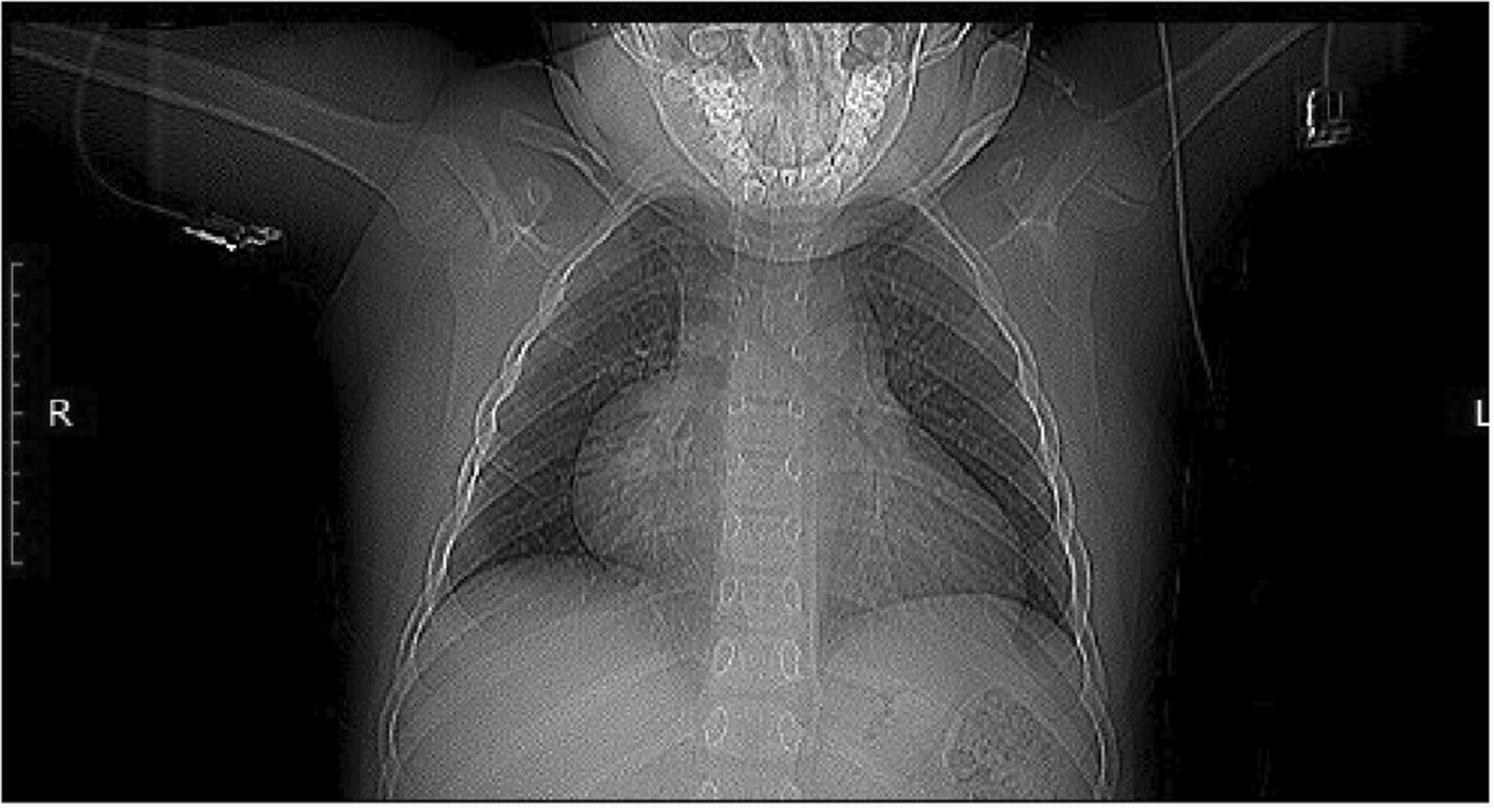
Pre-operative chest X-ray

The patient’s legal representative has signed an informed voluntary agreement for the use of medical data for scientific purposes. An 8-year-old boy weighing 45 kg was admitted to the Federal State Institution “Meshalkin National Medical Research Center” with supraventricular tachycardia and complains of fatigue despite minimal activity. The chest X-ray data indicated the presence of cardiomegaly, a condition characterised by enlargement of the right heart (Fig. 1).

During the echocardiography, an expansion of the RA cavity was identified on the anterolateral surface, which revealed the presence of a formation (*S* = 42 cm^2^, V up to 141 ml) with a connection to a RA measuring 4.1 cm in size. Concomitantly, the LV ejection fraction was within the normal range, amounting to 58%, with an LV EDV of 62 ml. A computed tomography (CT) scan and echocardiography (ECHO) were conducted to substantiate the diagnosis.

**Fig. 2:**
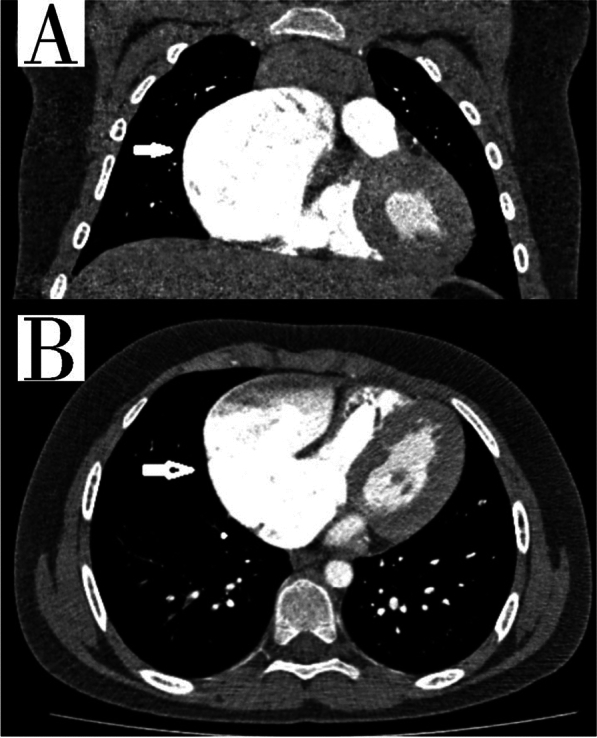
CT scan of the chest organs. **A** Frontal section, the arrow shows the aneurysmal expansion of the RA, **B** axial section, the arrow shows the aneurysmal expansion of the RA

**Fig. 3:**
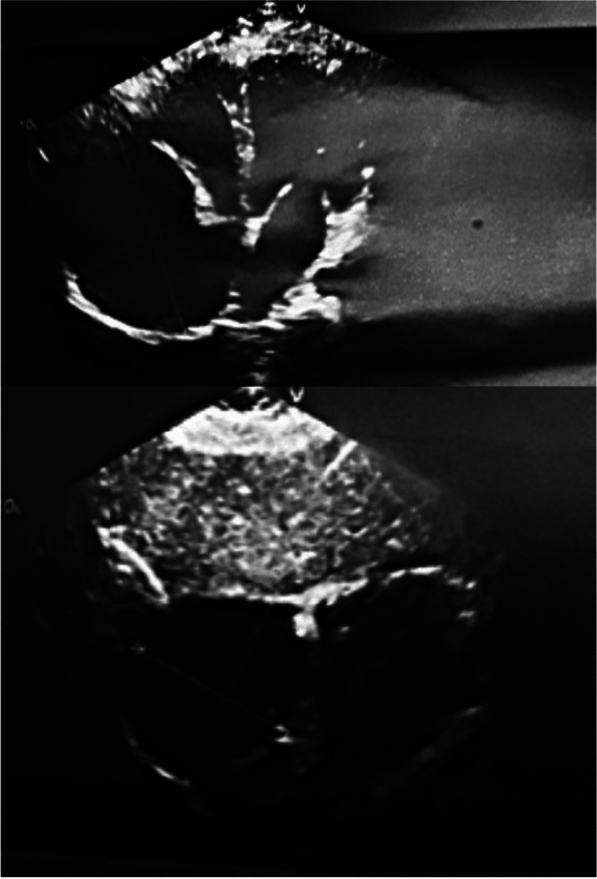
Pre-operative ECHO

According to CT and ECHO data, an aneurysm of the RA’s auricle with dimensions of 81 × 61x82 mm was identified (Figs. 2, 3).

**Fig. 4:**
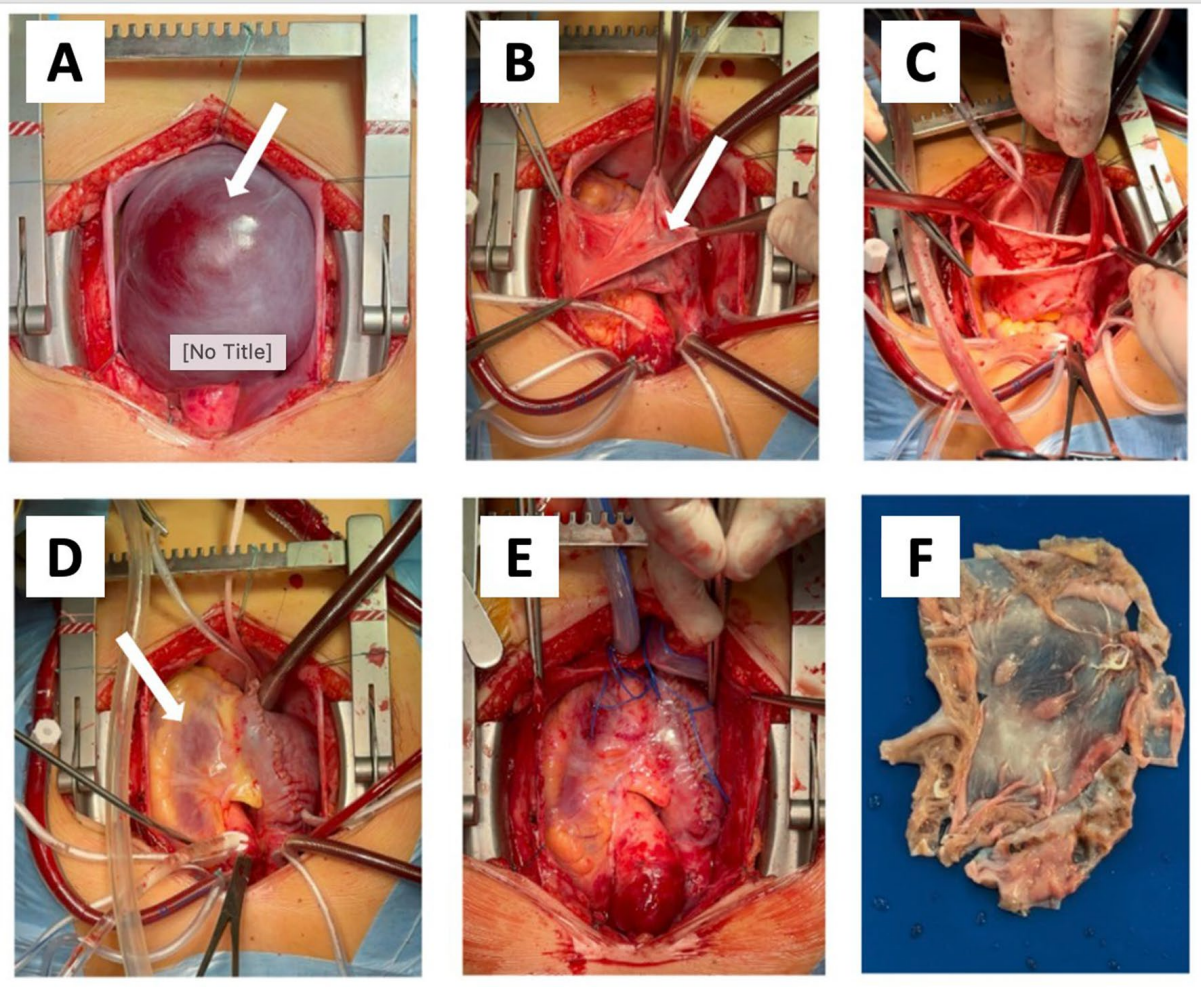
Intraoperative view of RA aneurysm correction. **A**, **B** View after median sternotomy, RA aneurysm is visualized, the arrow shows the thinned free wall of the RA, **C** resection of the thinned RA wall, **D** suturing RA. The arrow shows the deformation of the wall of the left ventricle resulting from compression of the RA aneurysm, **E** the heart after correction, **F** a macropreparation of a thinned RA wall

**Fig. 5:**
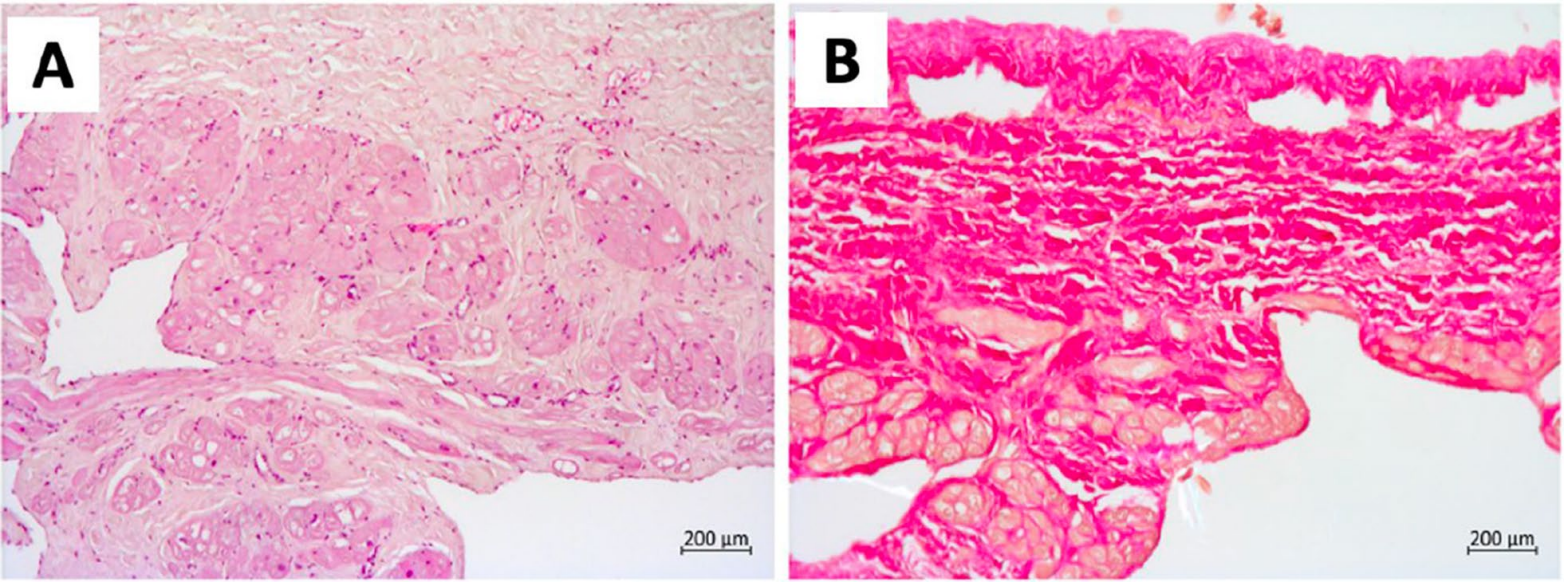
Microscopic preparation of the aneurysmal wall. A. This sample demonstrates a reduction in the thickness of the myocardium, with instances of complete absence. The muscle fibres display pronounced hypertrophy and dystrophy, manifesting as oedema, vacuolation of the cytoplasm, contractual changes and diffuse overgrowth of connective tissue. The endocardium is observed to be thin and uniform in consistency. A focal infiltration of lymphoid tissue is evident in the epicardium. The atrial wall displays a reduction in myocardial thickness, with complete absence in certain areas. Preserved muscle fibres with pronounced hypertrophy and dystrophy, manifesting as oedema, vacuolation of the cytoplasm, contractural changes and diffuse proliferation of connective tissue. The endocardium is observed to be thin and uniform in consistency. Focal lymphoid infiltration is observed in the epicardium. (**A**) Haematoxylin and eosin staining, (**B**) Van Gieson’s stain

**Fig. 6:**
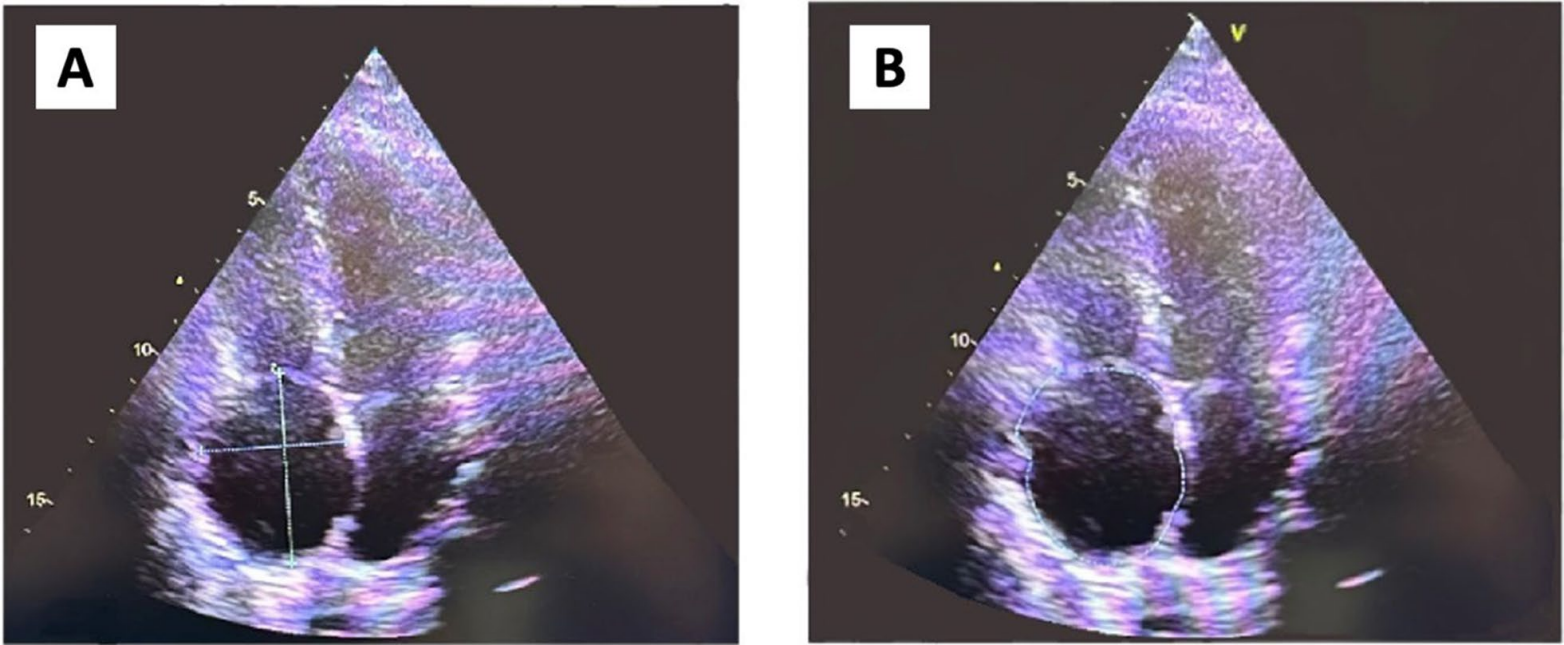
Postoperative TEE. **A** The lines indicate the short and long axis of the RA, **B** the line indicates the area of the RA

As illustrated in Fig. 2, CT data revealed the presence of an aneurysm in the auricle of the right atrium, measuring 81 × 61 × 82 mm in size. In the light of the CT data and the elevated risk of life-threatening arrhythmia and pulmonary embolism, the decision was taken to proceed with surgical correction in the form of resection of the RA aneurysm. A median sternotomy was performed, and a giant RA aneurysm (10 × 8 cm) was visualised (Fig. 4A). Bicaval cannulation is illustrated in Fig. 4b. An occlusion of the aorta was performed. Cardioplegia was administered using Custodiol (Dr. Franz Kohler Chemie, Alsbach-Hähnlein, Germany) in the aortic root. The right atrial aneurysm was resected at the boundary of the muscular tissue (Fig. 4B). The right atrium was then sutured (Fig. 4G). The occlusion time was 13 min. The duration of artificial blood circulation (ABC) was 23 min. Following decannulation, an episode of supraventricular tachycardia was observed. Despite the administration of pharmacological agents, the sinus rhythm remained unstable. Therefore, the restoration of sinus rhythm required the use of external mechanical circulatory support (EMF) (Fig. 4D). The resected portion was submitted for histological examination (Fig. 4E), which revealed severe myocardial dystrophy of the RA wall in the presence of widespread cardiosclerosis and enlargement of muscle fibres (Fig. 5). A control transoesophageal echocardiogram (TEE) was performed in the postoperative period (Fig. 6). The RA was visualised, with an area of 17.4 cm^2^ (short axis = 4.0 cm, long axis = 5.3 cm). Furthermore, a comparison of the data with the preoperative TEE indicators revealed an increase in LVEF to 74.2%, with an EDV index of 72 ml. According to the electrocardiogram, the patient exhibited a persistent sinus rhythm. The postoperative course was uncomplicated, and the patient was discharged 10 days after surgery.

At the six-month follow-up, Holter monitoring revealed a persistent sinus rhythm. The echocardiogram demonstrated an ejection fraction of 67%, a EDV index of 76 ml, and a left atrial area of 17 cm^2^. Notably, the patient reported no complaints during this follow-up visit.

## Discussion

An aneurysm of the right atrium (RA) or idiopathic enlargement of the RA is defined as a protrusion of the auricle of the RA and represents an extremely rare pathology of the cardiovascular system. It may be classified within the group of both CHD and acquired heart defect [1]. The aetiology of congenital RA aneurysm remains unknown. Two hypotheses have been proposed to explain the formation of RA aneurysms. The first suggests that the expansion of the auricle is caused by an abnormal synthesis of structural proteins, including collagen, and dysplasia of the pectinate muscles of the heart. The second hypothesis posits that the development of an aneurysm may result from embryonic loss of myoblasts, which could be triggered by a viral infection or injury. Acquired aneurysms of the RA auricle typically result from prolonged pulmonary hypertension, coronary heart disease, and chronic heart failure, which collectively lead to an overload of the RA volume.

Diagnosis of this defect is challenging and may be incidental during routine screening, given its prolonged asymptomatic course. In most cases, the first manifestation of clinical symptoms occurs in adulthood. However, there are instances where symptoms emerge during the neonatal period or in childhood, as documented in the literature [2–5]. Transoesophageal echocardiography is the primary instrumental diagnosis method. However, visualising an RA aneurysm with TEE is not always feasible. Therefore, CT angiography is the gold standard for establishing a definitive diagnosis due to its high sensitivity and specificity [2, 6]. 

To date, there is no optimal treatment method for aneurysmal enlargement of RA, which is the subject of considerable debate and discussion among cardiac surgery centres. It is proposed that conservative tactics should be employed in the treatment of RA aneurysms in patients with an asymptomatic course. This would entail the prescription of low-dose anticoagulants and antiarrhythmic drugs, with the aim of preventing the development of thrombosis and rhythm disturbances. Furthermore, such patients should limit their physical activity in order to prevent the rupture of the RA aneurysm. Nevertheless, the majority of cardiac surgeons are committed to surgical correction of this defect, both in cases where there is an asymptomatic course and in cases where there is a clinical presentation. This is because, in the absence of surgical treatment, there is a risk of developing various rhythm disturbances, pulmonary embolism, rupture of the RA, and sudden cardiac arrest.

In the clinical case presented, the medication was ineffective, as the patient had frequent progressive supraventricular tachycardia at the time of aneurysm detection, which became an indication for surgical correction. Furthermore, at the time of induction, ensuring surgical access, this patient exhibited a cardiac rhythm failure that was refractory to pharmacological intervention and necessitated electrical cardioversion. Therefore, the decision to pursue a radical correction of the RA aneurysm through surgical intervention represents the optimal course of action for achieving successful correction and improving the quality of life of this patient.

Currently, two approaches are available for the surgical treatment of an aneurysm of RA: aneurysmectomy, which is comparable to cardiopulmonary bypass (CPB) conditions, and the Josh and colleagues’ method, which involves the application of tactics similar to those used in CPB. This method involves the resection of RA aneurysms in the absence of CPB through the imposition of a vascular clamp on the RA wall. Nevertheless, the majority of cardiac surgery centres tend to opt for surgical correction under CPB conditions, given that this technique is deemed to be safer for patients with a thinned aneurysmal sac wall of varying sizes [1]. In our centre, a radical correction was performed in the volume of aneurysmectomy in CPB conditions, as the size of the aneurysm and the patient’s frequent cardiac arrhythmias precluded the use of the technique proposed by Josh and his colleagues.

## Conclusion

An aneurysm of the RA is an extremely rare cardiac anomaly with a variable clinical presentation. The aetiology of this disease remains unknown. In most cases, a RA aneurysm is diagnosed in children during preventive examinations or when a prominent clinical presentation emerges. Despite the availability of a conservative treatment approach, surgical correction remains the preferred option at the majority of cardiac surgery centres. This is not only to prevent the development of complications but also to achieve successful correction of RA aneurysms.

## Data Availability

No datasets were generated or analysed during the current study.

## Abbreviations

CHD: Congenital heart diseases
RV: Right ventricle
PE: Pulmonary embolism
CT: Computed tomography
CPB: Cardiopulmonary bypass
RA: Right atrium
